# Double-passage ground-state cooling induced by quantum interference in the hybrid optomechanical system

**DOI:** 10.1038/s41598-018-32719-1

**Published:** 2018-09-24

**Authors:** Lingchao Li, Ren-Hua Luo, Longjiang Liu, Shuo Zhang, Jian-Qi Zhang

**Affiliations:** 1 College of Science, Henan University of Technology, Zhengzhou, 450001 China; 2 Hubei Electric Engineering Corporation (POWERCHINA HEEC), Wuhan, 430040 China; 3 Zhengzhou Information Science and Technology Institute, Zhengzhou, 450004 China; 4 State Key Laboratory of Magnetic Resonance and Atomic and Molecular Physics, Wuhan Institute of Physics and Mathematics, Chinese Academy of Sciences, and Wuhan National Laboratory for Optoelectronics, Wuhan, 430071 China

## Abstract

We propose a quantum interference cooling scheme for a nano-mechanical resonator (NAMR) in a hybrid optomechanical system. In our scheme, atoms are trapped in an optomechanical cavity, and this optomechanical cavity interacts both atoms and an optical cavity. Therefore, the absorption of the optomechanical resonator can be modified by quantum interference effects induced by the atom-cavity and cavity-cavity couplings. With the modification of the quantum interference, the desired transition for cooling is enhanced, while the undesired transition for heating can be suppressed. As a result, the NAMR vibration can be cooled down to its ground state. Particularly, with the assistance of the atoms, the experimental difficulty can be reduced since the effective decay rate of the cavity can be decreased via the quantum interference for the atom-cavity coupling.

## Introduction

Cavity optomechanics works as an ideal platform to study the quantum properties of macroscopic mechanical systems. For this reason, it has been employed to create non-classical states^1,2^, realize quantum information processing^3–9^, and achieve precision control and measurement^10–13^. Although the NAMR is very sensitive to small deformation, and the precision measurement based on NAMR can approach to the Heisenberg limit^14^, the precision measurement is limited by the thermal noise on the NAMR. To further enhance the measurement sensibility, it is necessary to eliminate the thermal noise and cool the NAMR down to its ground state.

Until now, many different cooling schemes have been proposed to achieve the ground-state cooling of NAMR^15–18^. The most famous cooling method is the sideband cooling^19–21^, which works in the resolved-sideband regime, where the decay rates of the system is much less than the vibrational frequency of the NAMR, and it has been verified experimentally^22^. However, as the decay rates of the systems are always larger than the vibrational frequency of the NAMR, the sideband cooling is hard to be realized in the most of physical systems with NAMRs.

As such, considerable efforts have been made in the nonresolved-sideband regime, in which the decay rates would be larger than the vibrational frequency of the NAMR. For example, this kind of cooling can be realized with a dissipative coupling^23–27^, where the main dissipation is employed as the coupling between the cavity and the NAMR, and a fast ground-state cooling of the NAMR is available with time-dependent optical driven cavities^28^, where the large decay rates are employed to drive the cavities. The essential cooling method in the nonresolved-sideband regime is based on quantum interference^29–32^, in which the quantum interference is used to modify the absorption spectrum of the NAMR, and the NAMR can be cooled to its the ground state. The modification of the absorption spectrum is due to destructive interference of quantum noise^24,25^. Since there are large decay rates in the cooling schemes via quantum interference, the speed for quantum interference cooling can be much faster than the one for sideband cooling^33^.

On the other hand, with the development of the optomechanics^34^, the field of hybrid atom-optomechanics becomes an essential branch of the optomechanics. With the assistance of an additional atom, the quantum features of the optomechanic can be adjusted^35,36^, and atom-mechanical entanglement and quantum steering can also be achieved in this system^37–39^. Moreover, it has been shown theoretically and experimentally that the ground-state cooling of NAMR can be realized in hybrid atom-optomechanical systems^40–42^, as the absorption spectrum of the NAMR can be modified by the interaction between the atoms and the optical field indirectly, which allows the NAMR to be cooled down to its vibrational ground state.

Here we propose a cooling scheme with atoms trapped in an optomechanical cavity, which couples to a single-mode optical cavity. In our system, there are two different channels for quantum interference effects, one is from the atom-cavity coupling and the other is from the cavity-cavity coupling. Both of them satisfy the conditions for two-photon resonance. The combination of these two quantum interference effects can not only reduce the experimental difficult on the cavity quality, but also enhance the transition for cooling and suppress the one for heating. As a result, the ground-state cooling of the NAMR can be achieved.

Compared with the previous works involving only one quantum interference effect^43–45^, our scheme with the additional quantum interference effect is more efficient and can cool the NAMR down to its ground state with less mean phonon number. Moreover, different from the previous works for cooling, which is limited by the decay rates of the cavity^43,44^ and the atoms^45^, our scheme can work even with larger decay rates due to the combination of two quantum interference effects. As a result, our scheme reduces the experimental difficulty and works within a broader parameter regime.

## Results

### Model, Hamiltonian

**Figure 1:**
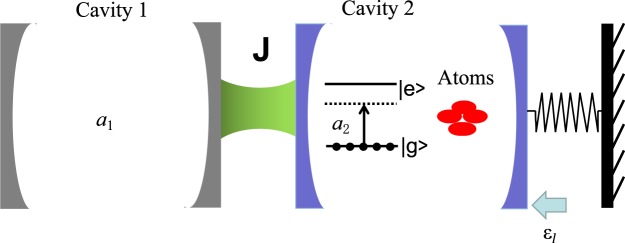
Schematic of our scheme. The optical cavity 1 couples to the optomechanical cavity 2 with the strength *J*. One two-level atomic ensemble, initially populated in its ground state, is trapped in the optomechanical cavity with a coupling strength *g*_*a*_. The optomechanical cavity is also driven by an external field *ε*_*l*_. The right-hand side mirror of the optomechanical cavity can be moved via radiation pressure.

As shown in Fig. 1, an ensemble of *N* atoms are trapped in an optomechanical cavity 2 with an atom-cavity coupling strength *g*_*a*_^46^. The optomechanical cavity 2 couples to a single-mode cavity 1 with a strength *J*, and is also driven by an external field at a frequency *ω*_*l*_ with a driven strength *ε*. Thus the Hamiltonian of this system can be written as (*ħ* = 1)

1 $$\begin{array}{rcl}H & = & {\omega }_{1}{a}_{1}^{\dagger }{a}_{1}+{\omega }_{2}{a}_{2}^{\dagger }{a}_{2}+{\omega }_{m}{b}^{\dagger }b+{\omega }_{eg}{S}_{ee}+i(\varepsilon {a}_{2}^{\dagger }{e}^{-i{\omega }_{{l}^{t}}}-{\varepsilon }^{\ast }{a}_{2}{e}^{i{\omega }_{{l}^{t}}})\\  &  & +\,J({a}_{1}^{\dagger }{a}_{2}+{a}_{2}^{\dagger }{a}_{1})+{g}_{a}({a}_{2}{S}_{eg}+{S}_{ge}{a}_{2}^{\dagger })-g{a}_{2}^{\dagger }{a}_{2}(b+{b}^{\dagger }),\end{array}$$

where *a*_*j*_ (${a}_{j}^{\dagger }$, *j* = 1, 2) and *b* $({b}^{\dagger })$ are annihilation (creation) operators for cavity *j* and NAMR, which take the corresponding frequencies *ω*_*j*_ and *ω*_*m*_, respectively, each atom in the ensemble owns an excited state $|e\rangle$ and a ground state $|g\rangle$ with a transition frequency *ω*_*eg*_, ${S}_{eg}={\sum }_{\mu \mathrm{=1}}^{N}\,|{e}^{\mu }\rangle \langle {g}^{\mu }|$ and ${S}_{ee}={\sum }_{\mu \mathrm{=1}}^{N}\,|{e}^{\mu }\rangle \langle {e}^{\mu }|$; *g* is the single-photon radiation coupling coefficient. The first four items in Hamiltonian (1) are for the free Hamiltonians of the two cavities, the NAMR and the atoms, respectively. The fifth item shows the driven field on the optomechanics. The last items describe the interactions for cavity-cavity, atom-cavity, and radiation coupling, respectively.

Assume the atom number N of the atomic ensemble is large enough, under the condition of the weak excitation, we can define the ground and excited state of the atomic ensemble as |***g***〉 = |*g*_1_, *g*_2_, ..., *g*_*N*_〉 and $|{\bf{e}}\rangle =\frac{1}{\sqrt{N}}{\Sigma }_{j\mathrm{=1}}^{N}\,|{g}_{1},{g}_{2},,{e}_{j},\,\ldots ,\,{g}_{N}\rangle$, respectively. Then the above Hamiltonian (1) can be rewritten as

2 $$\begin{array}{rcl}H & = & {\omega }_{1}{a}_{1}^{\dagger }{a}_{1}+{\omega }_{2}{a}_{2}^{\dagger }{a}_{2}+{\omega }_{m}{b}^{\dagger }b+{\omega }_{eg}{S}_{ee}+i(\varepsilon {a}_{2}^{\dagger }{e}^{-i{\omega }_{{l}^{t}}}-{\varepsilon }^{\ast }{a}_{2}{e}^{i{\omega }_{{l}^{t}}})\\  &  & +\,J({a}_{1}^{\dagger }{a}_{2}+{a}_{2}^{\dagger }{a}_{1})+\sqrt{N}{g}_{a}({a}_{2}{S}_{eg}+{S}_{ge}{a}_{2}^{\dagger })-g{a}_{2}^{\dagger }{a}_{2}(b+{b}^{\dagger }),\end{array}$$

with *S*_*ee*_ = |***e***〉〈***e***| and *S*_*eg*_ = |***e***〉〈***g***|.

Define the detuning Δ_1,2_ = *ω*_1,2_ − *ω*_*l*_ and Ω = *ω*_*eg*_ − *ω*_*l*_ and assume $({g}_{a}\sqrt{N},{g}_{a}\langle {a}_{2}\rangle )\ll |{\rm{\Omega }}-{{\rm{\Delta }}}_{2}|$, we can ensure that only one of the atom in the atomic ensemble can be virtual excited^37,47^. In the rotating frame with frequency *ω*_*l*_, the Hamiltonian (2) can be rewritten as

3 $$\begin{array}{rcl}H & = & {{\rm{\Delta }}}_{1}{a}_{1}^{\dagger }{a}_{1}+{{\rm{\Delta }}}_{2}{a}_{2}^{\dagger }{a}_{2}+{\omega }_{m}{b}^{\dagger }b+{\rm{\Omega }}{S}_{ee}+i(\varepsilon {a}_{2}^{\dagger }-{\varepsilon }^{\ast }{a}_{2})\\  &  & +\,J({a}_{1}^{\dagger }{a}_{2}+{a}_{2}^{\dagger }{a}_{1})+{g}_{a}\sqrt{N}({a}_{2}{S}_{eg}+{S}_{ge}{a}_{2}^{\dagger })-g{a}_{2}^{\dagger }{a}_{2}(b+{b}^{\dagger }\mathrm{).}\end{array}$$

The dynamics of this system is governed by following quantum Langevin equations,

4 $$\begin{array}{rcl}{\dot{a}}_{1} & = & -({\kappa }_{1}+i{{\rm{\Delta }}}_{1}){a}_{1}-iJ{a}_{2}+\sqrt{2{\kappa }_{1}}{a}_{\mathrm{1,}in},\\ {\dot{a}}_{2} & = & -({\kappa }_{2}+i{{\rm{\Delta }}}_{2}){a}_{2}-iJ{a}_{1}-i\sqrt{N}{g}_{a}{S}_{ge}+ig{a}_{2}(b+{b}^{\dagger })+\varepsilon +\sqrt{2{\kappa }_{2}}{a}_{\mathrm{2,}in},\\ \dot{b} & = & -({\gamma }_{m}+i{\omega }_{m})b+ig{a}_{2}^{\dagger }{a}_{2}+\sqrt{2{\gamma }_{m}}{b}_{in},\\ {\dot{S}}_{ge} & = & -(\gamma +i{\rm{\Omega }}){S}_{ge}-i\sqrt{N}{g}_{a}{a}_{2}+\sqrt{2\gamma }{f}_{in},\end{array}$$

where *κ*_*j*_, *γ*_*m*_ and *γ* denote the decay rates of the cavity *j*, NAMR and atomic ensemble, respectively. *O*_*in*_ is the noise operator with a nonzero correlation function $\langle {O}_{in}(t){O}_{in}^{\dagger }(t^{\prime} )\rangle =\delta (t-t^{\prime} )$, for *O* = *a*_1,2_, and *f*. *b*_*in*_ is the quantum Brownian noise operator of the NAMR with correlation functions $\langle {b}_{in}^{\dagger }(t){b}_{in}(t^{\prime} )\rangle ={n}_{m}\delta (t-t^{\prime} )$, and $\langle {b}_{in}(t){b}_{in}^{\dagger }(t^{\prime} )\rangle =({n}_{m}+\mathrm{1)}\delta (t-t^{\prime} )$, where *n*_*m*_ = [exp(*ω*_*m*_/(*k*_*B*_*T*)) − 1]^−1^ is the thermal phonon number with *k*_*B*_ and *T* being the Boltzmann constant and the environment temperature, respectively.

Suppose that the atomic ensemble is initially in its the ground state $|{\bf{g}}\rangle$^48^. Then the steady-state values of the system are

$$\langle {a}_{1}\rangle =-\frac{iJ\langle {a}_{2}\rangle }{{\kappa }_{1}+i{{\rm{\Delta }}}_{1}}\mathrm{,\ }\langle {a}_{2}\rangle =\frac{\varepsilon }{{\kappa }_{2}+i{\tilde{{\rm{\Delta }}}}_{2}+\frac{{g}_{a}^{2}N}{\gamma +i{\rm{\Omega }}}+\frac{{J}^{2}}{{\kappa }_{1}+i{{\rm{\Delta }}}_{1}}},$$

$$\langle b\rangle =\frac{ig{|\langle {a}_{2}\rangle |}^{2}}{{\gamma }_{m}+i{\omega }_{m}},\langle {S}_{ge}\rangle =\frac{i{g}_{a}N\langle {a}_{2}\rangle }{\gamma +i{\rm{\Omega }}},$$

with ${\tilde{{\rm{\Delta }}}}_{2}={{\rm{\Delta }}}_{2}-g(\langle b\rangle +\langle {b}^{\dagger }\rangle )$. In the case of *g*_*a*_ = 0, our system would be reduced to the model in ref.^43^.

Under the condition of the weak excitation, only one atom can be virtual excited, and the rest atoms are in ground state^37,47^. With the application of the linearization approximation^18,19^, all the operators can be written as the sum of steady-state mean values and their fluctuations, e.g., *a*_1,2_ = 〈*a*_1,2_〉 + *δa*_1,2_, *b* = 〈*b*〉 + *δb*, *S*_*ge*_ = 〈*S*_*ge*_〉 + *δS*_*ge*_. Then the above Langevin equations are rewritten as

5 $$\begin{array}{rcl}\delta {\dot{a}}_{1} & = & -({\kappa }_{1}+i{{\rm{\Delta }}}_{1})\delta {a}_{1}-iJ\delta {a}_{2}+\sqrt{2{\kappa }_{1}}{a}_{\mathrm{1,}in},\\ \delta {\dot{a}}_{2} & = & -({\kappa }_{2}+i{\tilde{{\rm{\Delta }}}}_{2})\delta {a}_{2}-iJ\delta {a}_{1}-i\sqrt{N}{g}_{a}\delta {S}_{ge}+iG(\delta b+\delta {b}^{\dagger })+\sqrt{2{\kappa }_{2}}{a}_{\mathrm{2,}in},\\ \delta \dot{b} & = & -({\gamma }_{m}+i{\omega }_{m})\delta b+iG(\delta {a}_{2}^{\dagger }+\delta {a}_{2})+\sqrt{2{\gamma }_{m}}{b}_{in},\\ \delta {\dot{S}}_{ge} & = & -(\gamma +i{\rm{\Omega }})\delta {S}_{ge}-i\sqrt{N}{g}_{a}\delta {a}_{2}+\sqrt{2\gamma }{f}_{in},\end{array}$$

with *G* = *g*〈*a*_2_〉.

The corresponding effective Hamiltonian for the above Langevin equations can be described by

$${H}_{eff}={H}_{a-c}+{\omega }_{m}\delta {b}^{\dagger }\delta b-g(\delta {a}_{2}^{\dagger }+\delta {a}_{2})(\delta b+\delta {b}^{\dagger })$$

with

6 $$\begin{array}{rcl}{H}_{a-c} & = & {{\rm{\Delta }}}_{1}\delta {a}_{1}^{\dagger }\delta {a}_{1}+{\tilde{{\rm{\Delta }}}}_{2}\delta {a}_{2}^{\dagger }\delta {a}_{2}+{\rm{\Omega }}\delta {S}_{ee}\\  &  & +\,J(\delta {a}_{1}^{\dagger }\delta {a}_{2}+\delta {a}_{2}^{\dagger }\delta {a}_{1})+\sqrt{N}{g}_{a}(\delta {a}_{2}\delta {S}_{eg}+\delta {S}_{ge}\delta {a}_{2}^{\dagger }\mathrm{).}\end{array}$$

### The rate equations for cooling

Following the method in refs^19,49^, the rate equation for the phonon on the NAMR is given by

7 $$\begin{array}{rcl}{\dot{P}}_{n} & = & ({A}_{-}+{\gamma }_{m}({n}_{m}+\mathrm{1)})(n+\mathrm{1)}{P}_{n+1}+({A}_{+}+{\gamma }_{m}{n}_{m})n{P}_{n-1}\\  &  & -[{A}_{-}n+{A}_{+}(n+\mathrm{1)}+{\gamma }_{m}({n}_{m}+\mathrm{1)}n+{\gamma }_{m}{n}_{m}(n+\mathrm{1)}]{P}_{n},\end{array}$$

where *P*_*n*_ is the probability for the NAMR in the Fock state $|n\rangle$. *A*_−_ = *G*^2^*S*_*FF*_(*ω*_*m*_) is the cooling rate of the NAMR while *A*_+_= *G*^2^*S*_*FF*_(−*ω*_*m*_) is for the heating rate, and ${S}_{FF}(\omega )=\int dt{e}^{i\omega t}\langle F(t)F\mathrm{(0)}\rangle$ is the absorption spectrum for the radiation force $F=\delta {a}_{2}^{\dagger }+\delta {a}_{2}$.

To obtain the absorption spectrum, we write the Langevin equations of Hamiltonian (6) for cooling as follows

8 $$\begin{array}{rcl}\delta {\dot{a}}_{1} & = & -({\kappa }_{1}+i{{\rm{\Delta }}}_{1})\delta {a}_{1}-iJ\delta {a}_{2}+\sqrt{2{\kappa }_{1}}{a}_{\mathrm{1,}in},\\ \delta {\dot{a}}_{2} & = & -({\kappa }_{2}+i{\tilde{{\rm{\Delta }}}}_{2})\delta {a}_{2}-iJ\delta {a}_{1}-i\sqrt{N}{g}_{a}\delta {S}_{ge}+\sqrt{2{\kappa }_{2}}{a}_{\mathrm{2,}in},\\ \delta {\dot{S}}_{ge} & = & -(\gamma +i{\rm{\Omega }})\delta {S}_{ge}-i\sqrt{N}{g}_{a}\delta {a}_{2}+\sqrt{2\gamma }{f}_{in}\mathrm{.}\end{array}$$

In the weak coupling regime, the back action of the NAMR can be ignored. So the absorption spectrum *S*_*FF*_(*ω*) of the radiation force can be calculated from the quantum Langevin Eq. (8) as

9 $${S}_{FF}(\omega )=\frac{1}{B(\omega )}+\frac{1}{{B}^{\ast }(\omega )}=\frac{1}{|B(\omega {)|}^{2}}(2{\kappa }_{2}+\frac{2{J}^{2}{\kappa }_{1}}{|{\kappa }_{1}-i(\omega -{{\rm{\Delta }}}_{1}{)|}^{2}}+\frac{2N{g}_{a}^{2}\gamma }{|\gamma -i(\omega -{\rm{\Omega }}{)|}^{2}}),$$

with

10 $$B(\omega )={\kappa }_{2}-i(\omega -{\tilde{{\rm{\Delta }}}}_{2})+\frac{{J}^{2}}{{\kappa }_{1}-i(\omega -{{\rm{\Delta }}}_{1})}+\frac{N{g}_{a}^{2}}{\gamma -i(\omega -{\rm{\Omega }})}.$$

The three items in Eq. (9) correspond to the correlation functions of the fluctuations from the cavity 2, cavity 1, and atoms, respectively.

With the application of the rate Eq. (7), the final mean phonon number of the NAMR is

11 $$\begin{array}{rcl}{n}_{f} & = & \frac{{\gamma }_{m}{n}_{m}+{\gamma }_{c}{n}_{c}}{{\gamma }_{m}+{\gamma }_{c}}\\  & \simeq  & \frac{{\gamma }_{c}{n}_{c}}{{\gamma }_{m}+{\gamma }_{c}}({\gamma }_{m}{n}_{m}\ll {\gamma }_{c}{n}_{c})\end{array},$$

where

$${\gamma }_{c}={A}_{-}-{A}_{+},$$

is the extra damping rate of the mechanical oscillator,

$${n}_{c}=\frac{{A}_{+}}{{A}_{-}-{A}_{+}},$$

is the final mean phonon number under the ideal condition.

### Absorption spectrums and cooling processes

Now, we will discuss the absorption spectrum and the cooling processes in our system.

**Figure 2:**
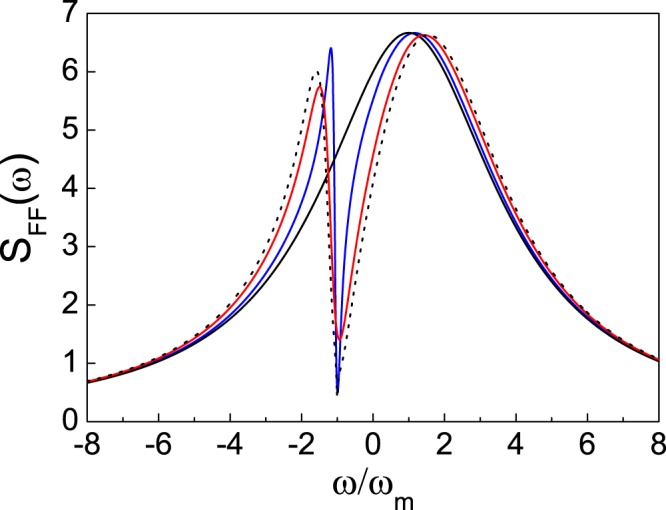
Optical fluctuation spectrum *S*_*FF*_(*ω*) versus the frequency *ω* for the coupling coefficients (*J*, *g*_*a*_), with black solid line (*J* = 0, *N* = 0), red solid line (*J* = *ω*_*m*_, *N* = 0), blue solid line (*J* = 0, ${g}_{a}\sqrt{N}=0.6{\omega }_{m}$), and black dotted line (*J* = *ω*_*m*_, ${g}_{a}\sqrt{N}=0.6{\omega }_{m}$). The effective detuning of the first cavity mode Δ_1_ = Ω = −*ω*_*m*_ with the decay rate *κ*_1_ = 0.1 *ω*_*m*_, while the second cavity mode ${\tilde{{\rm{\Delta }}}}_{2}$ = *ω*_*m*_ with its decay rate *κ*_2_ = 3 *ω*_*m*_. The atomic decay rate is *γ* = 0.01 *ω*_*m*_.

The absorption spectrum *S*_*FF*_(*ω*) versus the frequency *ω* with different coupling strengths (*J*, *g*_*a*_) are plotted in Fig. 2. Since the optomechanical cavity works in the nonresolved-sideband regime, i.e., the decay rate of cavity is much larger than the frequency of the NAMR, when the optomechanical cavity 2 is decoupled from the cavity 1 and the atom ensemble (*J* = 0, *N* = 0), the absorption spectrum is in a Lorentz profile with a half width *κ*_2_. On the other hand, with the assistance of the good cavity and the atomic ensemble, the Lorentz profile can be modified to a Fano one for quantum interference^50,51^, and the ground-state cooling of the NAMR can be achieved for this reason.

Quantum interference can be followed from the eigen-energies of the dressed states of Hamiltonian (6)

12 $$\begin{array}{rcl}{E}_{\pm } & = & \frac{1}{2}({\rm{\Omega }}+{\tilde{{\rm{\Delta }}}}_{2}\pm \sqrt{\mathrm{4(}{J}^{2}+{g}_{a}^{2}N)+{({\rm{\Omega }}-{\tilde{{\rm{\Delta }}}}_{2})}^{2}}),\\ {E}_{0} & = & {\rm{\Omega }},\end{array}$$

here we have set Δ_1_ = Ω. These three eigen-energies correspond to three inflection points in the absorption spectrum *S*_*FF*_(*ω*) [see Fig. 2]. *E*_0_ = Ω denotes a dark state, meaning no absorption at this point for the quantum destructive interference. As a result, we can set

13 $${{\rm{\Delta }}}_{1}={\rm{\Omega }}=-\,{\omega }_{m}$$

to suppress the transition for heating process. Moreover, to get the ground-state cooling of the NAMR, the value *S*_*FF*_(*ω* = *ω*_*m*_) in the absorption spectrum for cooling must be enhanced. In other words, it is necessary to ensure *E*_+_= *ω*_*m*_, so that the absorption spectrum *S*_*FF*_(*ω* = *ω*_*m*_) can reach its maximum value by adjusting the coupling strengths (*J*, *g*_*a*_) with quantum constructive interference. Then, we get the optimal condition for two coupling strengths as

14 $${J}^{2}+{g}_{a}^{2}N=2{\omega }_{m}({\omega }_{m}-{\tilde{{\rm{\Delta }}}}_{2})\geqslant 0,$$

which is independent of the decay rates, since the eigen-energy has no relation with the decay rates.

**Figure 3:**
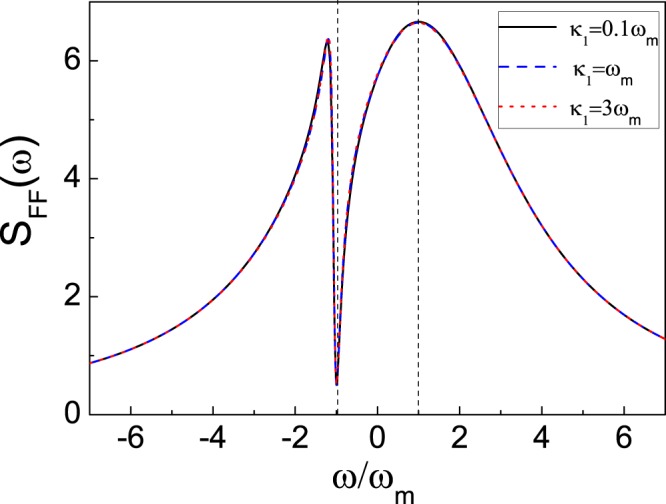
Optical fluctuation spectrum *S*_*FF*_(*ω*) versus *ω* for different decay rates, i.e., *κ*_1_ = 0.1 *ω*_*m*_ (solid line), *κ*_1_ = *ω*_*m*_ (dashed line) and ${\kappa }_{1}=3{\omega }_{m}$ (dotted line). Other parameters are given by ${g}_{a}\sqrt{N}=0.6{\omega }_{m}$, *γ* = 0.01 *ω*_*m*_, *κ*_2_ = 3 *ω*_*m*_, ${\tilde{{\rm{\Delta }}}}_{2}$ = 0.8 *ω*_*m*_, Δ_1_ = −*ω*_*m*_.

Figure 3 shows the absorption spectrum *S*_*FF*_(*ω*) versus *ω* for the decay rate *κ*_1_ of the cavity *a*_1_ with parameters of ${\tilde{{\rm{\Delta }}}}_{2}$ = −0.1 *ω*_*m*_ and *J* = 0.45 *ω*_*m*_. Under the optimal condition as Eq. (14), the heating process can be completely suppressed [i.e., the value of *S*_*FF*_(*ω* = −*ω*_*m*_) approaches zero], the absorption spectrum value *S*_*FF*_(*ω* = *ω*_*m*_) for the cooling process can reach its maximal value of the curve with a good cavity (*κ*_1_ = 0.1 *ω*_*m*_). With the increase of the cavity decay rate *κ*_1_, a ground-state cooling can still be achieved with a bad cavity (*κ*_1_ = 3 *ω*_*m*_), since the absorption spectrum for the bad cavity is almost same as the one for the good cavity. It results from the fact that the heating process is suppressed by the quantum interference for the atom-cavity coupling. As such, different from the methods in refs^43–45^, where the good cavity^43,44^ (atom^45^)is an essential condition to guarantee the minimal value *S*_*FF*_(−*ω*_*m*_) approaching zero, our scheme, which combines two quantum interference effects, can work beyond this condition.

**Figure 4:**
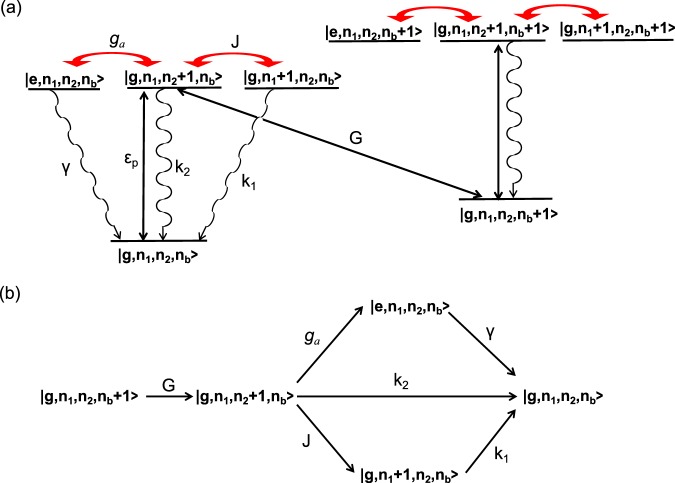
(**a**) Level scheme for the cooling mechanism and (**b**) the flowchart for cooling processes. Here $|{n}_{1}\rangle$, $|{n}_{2}\rangle$ and $|{n}_{b}\rangle$ denote the states of the optical and optomechanical cavity and the oscillator, respectively. $|g\rangle$ and $|e\rangle$ denote the atomic ground and excited states, respectively.

With the assistance of quantum interference, the cooling mechanism of the hybrid optomechanical system in our scheme can be understood from Fig. 4, where $|{n}_{1}\rangle$, $|{n}_{2}\rangle$ and $|{n}_{b}\rangle$ are the states of the single-mode cavity 1, optomechanical cavity 2 and the NAMR, respectively. If the system is initially in the state $|{\bf{g}},{n}_{1},{n}_{2},{n}_{b}\rangle$, under the action of the optical pump field, the photons are injected into the optomechanical cavity 2, the state $|{\bf{g}},{n}_{1},{n}_{2},{n}_{b}\rangle$ will evolve to the state $|{\bf{g}},{n}_{1},{n}_{2}+1,{n}_{b}\rangle$. In this situation, the photons in optomechanical cavity 2 are transferred into the phonons on the NAMR via the transition $|{\bf{g}},{n}_{1},{n}_{2}+1,{n}_{b}\rangle \to |{\bf{g}},{n}_{1},{n}_{2},{n}_{b}+1\rangle$ by the radiation coupling *G*. This cooling transition can be enhanced by two quantum constructive interference effects. One is between the two transitions $|{\bf{g}},{n}_{1},{n}_{2}+1,\,{n}_{b}\rangle$ → $|{\bf{g}},{n}_{1}+1,{n}_{2},{n}_{b}\rangle$ → $|{\bf{g}},{n}_{1},{n}_{2},{n}_{b}\rangle$ and $|{\bf{g}},{n}_{1},{n}_{2}+1,{n}_{b}\rangle$ → $|{\bf{g}},{n}_{1},{n}_{2},{n}_{b}\rangle$. The other is from the transitions $|{\bf{g}},{n}_{1},{n}_{2}+1,{n}_{b}\rangle$ → $|{\bf{e}},{n}_{1},{n}_{2},{n}_{b}\rangle$ → $|{\bf{g}},{n}_{1},{n}_{2},{n}_{b}\rangle$. These two quantum constructive interference effects can also enhance the transition from $|{\bf{g}},{n}_{1},{n}_{2},{n}_{b}+1\rangle$ to $|{\bf{g}},{n}_{1},{n}_{2}+1,{n}_{b}\rangle .$

### Numerical Simulation

Next, we will verify the cooling effect of our scheme with experimental parameters. These experimental parameters for simulations are the follows^52^: *ω*_*m*_ = 2*π* × 20 MHz, *Q*_*m*_ = *ω*_*m*_/*γ*_*m*_ = 8 × 10^4^, *g* = 1.2 × 10^−4^ *ω*_*m*_, |*ε*| = 600 *ω*_*m*_, *T* = 300 mK), Δ_1_ = Ω = −*ω*_*m*_ and *κ*_2_ = 3 *ω*_*m*_.

**Figure 5:**
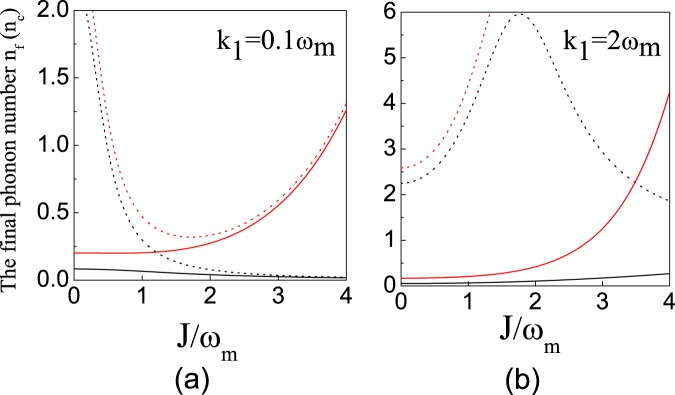
The final phonon number *n*_*c*_ (black line) with *T* = 0 *K* and the final phonon number *n*_*f*_ (red line) with *T* = 300 *mK* versus the coupling coefficient *J* for different decay rates of the auxiliary cavity (**a**) *κ*_1_ = 0.1 *ω*_*m*_ and (**b**) *κ*_1_ = 2 *ω*_*m*_. The dotted lines denote the pure optomechanical system, i.e., *N* = 0, and the solid lines denote the hybrid optomechanical system, i.e., ${g}_{a}\sqrt{N}=0.6{\omega }_{m}$. Other parameters are chosen as *κ*_2_ = 3 *ω*_*m*_, *γ* = 0.01 *ω*_*m*_. The effective detuning ${\tilde{{\rm{\Delta }}}}_{2}$ always satisfies the optimal condition of Eq. (14).

The final mean phonon numbers *n*_*c*_ (*n*_*f*_) versus the cavity-cavity coupling strength *J* in the resolved regime and non-resolved regime are simulated in Fig. 5(a,b), respectively. When the system works in the resolved regime, in the idea case (*T* = 0 K), with the increase of the cavity-cavity coupling strength *J*, the final mean phonon number *n*_*c*_ can be approach to zero [see Fig. 5(a)]. It’s due to the fact that, with the increase of the cavity-cavity coupling strength *J*, the quantum interfrence can enhance the line width of the spectrum for non-absorption, and suppress the 2nd-order transition for heating largely. Note that, when the atomic ensemble is decoupled from the optomechanical cavity $(\sqrt{N}{g}_{a}=0)$, our results return to the ones in refs^43,44^. Moreover, after the environmental heating is included, the final phonon number will be increased. It results from the fact that, with the increase of the cavity-cavity coupling strength *J*, the eigen-energy ${E}_{+}=\frac{1}{2}({\rm{\Omega }}+{\tilde{{\rm{\Delta }}}}_{2}+\sqrt{\mathrm{4(}{J}^{2}+{g}_{a}^{2}N)+{({\rm{\Omega }}-{\tilde{{\rm{\Delta }}}}_{2})}^{2}})$ will deviate from *E*_+_ ≡ *ω*_*m*_, and the cooling rate will decrease, while the environmental heating is a constant, and the final phonon number will increase.

It’s worthy to point out the follows. With the introduction of the atomic ensemble, our scheme can cool the NAMR to its ground state when the cavity-cavity coupling is very weak, since the additional quantum interference can be created by the atom-cavity coupling.

On the other hand, when the system works in the non-resolved regime, the quantum interference for the cavity-cavity coupling can’t cool the NAMR to its ground state, since the first blue side-band transition can’t be suppressed totally when the cavity decay rate is very large. However, this problem can be overcome by adding the atomic ensemble [see Fig. 5(b)]. In this situation, the additional quantum interference caused by the atoms can further modify the absorption spectrum, and the final mean phonon number *n*_*c*_ turns to be very close to *n*_*c*_ = 0 [see black line in Fig. 5(b)]. It means that the hybrid optomechanical system in our scheme can cool the NAMR to its ground state more effectively due to the combination of two quantum interference effects for the atom-cavity coupling and cavity-cavity coupling, and thus the experimental difficulties are reduced in this way.

## Discussion

In summary, we have shown the NAMR can be cooled to its ground state in the hybrid optomechanical system, where one two-level atomic ensemble is trapped in the optomechanical cavity, which is coupling to an additional optical cavity. Due to combination of two quantum interference effects from the atom-cavity coupling and cavity-cavity coupling, the heating processes are suppressed, while the cooling processes are enhanced. Compared with previous cooling methods involving only one quantum interference effect^43–45^, the combination of two quantum interference effects can reduce the limit on the line-widths of the cavity and atoms. As a result, our scheme can cool the NAMR down to its ground state more efficiently. In particular, our scheme is experimentally feasible for lower-quality cavities, and the experimental difficulty can be reduced in this way.
